# Percutaneous vertebral-disc plasty for thoracolumbar very severe osteoporotic vertebral compression fractures: A randomized controlled study

**DOI:** 10.3389/fsurg.2022.1010042

**Published:** 2022-10-19

**Authors:** Jiawei Jiang, Jinlong Zhang, Guofeng Bao, Jiajia Chen, Chunshuai Wu, Hongxiang Hong, Pengfei Xue, Guanhua Xu, Zhiming Cui

**Affiliations:** Department of Spine Surgery, The Affiliated 2 Hospital of Nantong University, Nantong, China

**Keywords:** percutaneous vertebroplasty, percutaneous cement discoplasty, very severe osteoporotic vertebral compression fractures, segmental instability, kyphosis

## Abstract

**Purpose:**

To compare the clinical outcomes and radiological parameters of patients undergoing percutaneous vertebroplasty (PVP) versus those undergoing percutaneous vertebral-disc plasty (PVDP) for back pain, segmental instability, and kyphosis due to thoracolumbar very severe osteoporotic vertebral compression fractures (vsOVCFs).

**Methods:**

This prospective randomized controlled study included elderly patients with thoracolumbar vsOVCFs. All the patients were randomly allocated into the PVP group (who underwent conventional PVP) and the PVDP group (who underwent PVP combined percutaneous cement discoplasty). The visual analogue scale (VAS), Oswestry Disability Index (ODI), local kyphosis angle, and disc height were recorded preoperatively and postoperatively.

**Results:**

Significant postoperative improvements in the VAS, ODI, and the local kyphosis angle (LKA) were shown, compared with the preoperative values in both groups (*p* < 0.05). The average VAS, ODI, and LKA for patients in the PVP group were increased compared to those in the PVDP group observed at the last follow-up (*p* < 0.05). The DHA, DHP, and LKA were seen to be maintained in the PVDP group at the last follow-up (*p* > 0.05). The change was significantly lower in the PVDP group at the last follow-up in those parameters (*p* < 0.05).

**Conclusion:**

PVDP may be a feasible and effective technique for the treatment of very severe OVCFs, that can restore intervertebral height, provide segmental stabilizing and relieve back pain in the short term.

## Introduction

Osteoporosis is a major global public health problem, causing more than 8.9 million fractures each year (1). Osteoporotic vertebral compression fracture (OVCF) is the most frequent type of fracture in the elderly (2). OVCF usually causes significant back pain and mobility limitations (3). The conservative treatments need prolonged bed rest and drug therapy (4). If patients are associated with a delay in accepting formal conservative treatment, multiple complications secondary to OVCF will occur, such as back pain, kyphotic deformity, a reduction of life quality, increased magnitude of vertebral compression, deep vein thrombosis, and pulmonary infection (5, 6).

Very severe osteoporotic vertebral compression fractures (vsOVCFs) have been defined as compression of the vertebral anterior column greater than two-thirds of their original height (7), which is accompanied by kyphosis deformity and endplate-disc complex injury (EDCI) (8, 9). Folman (10) proposed that the local kyphosis angle (LKA) exceeds 20°, and the compression of the anterior column of the diseased vertebral column exceeds 50%, which can be considered as local instability of the fracture. In patients with vsOVCFs, vertebral compression and EDCI are often aggravated due to the failure of formal conservative treatment in the early stage of the disease, which accelerates the degeneration of the damaged intervertebral disc and even the appearance of disc vacuum phenomenon, which further leads to instability of the thoracolumbar spine and further kyphotic deformity.

Traditional osteotomy and pedicle screw fixation and fusion operation are used to correct kyphotic deformity and treat segmental instability, which is too risky for elderly patients with severe underlying disease (11). As imaging and surgical techniques have improved, percutaneous vertebroplasty (PVP) has gradually been applied to the treatment of vsOVCFs that was once considered a relative contraindication to surgery (12), but the results are quite different. Varga et al. (13) introduced an emerging minimally invasive surgery called the percutaneous cement discoplasty (PCD), which was modified and supplemented by Sola et al. (14). PCD is used effectively to treat axial pain and disability associated with severe lumbar disc degeneration and provides a prompt segmental stabilizing effect. Nevertheless, this technology is not yet used in vsOVCFs. Because patients with vsOVCFs often suffer from residual back pain due to spinal instability after PVP, and the incidence of intervertebral disc cement leakage is particularly high, it is natural to link the two techniques together. Much like the terms vertebroplasty and kyphoplasty, we call the procedure percutaneous vertebral-disc plasty (PVDP).

We hypothesize that the injection of bone cement into the intervertebral disc is the critical solution for a better outcome for vsOVCFs. In this study, we performed this prospective analysis of clinical and radiological results in two patient groups with vsOVCFs, one treated with PVP and the other with PVDP. Our objective was to describe this minimally invasive technique and evaluate the feasibility, safety, and clinical efficacy of the treatment.

## Materials and methods

### Study design

This was a prospective randomized controlled trial in that patient diagnosed with thoracolumbar vsOVCFs in our hospital and undergoing minimally invasive treatment from November 2019 to March 2021 were enrolled for the study. The study was planned according to Consolidated Standards of Reported Trials (CONSORT) and approved by the Ethics Committee of the Affiliated 2 Hospital of Nantong University (ethics number is 2022KT196). Meanwhile, the clinical study registration number is NCT05519332 on ClinicalTrials.gov. Written consent was obtained from each participating patient before starting the operation.

### Study population

The patients were randomly allocated into two groups (based on random numbers generated by www.randomizer.org), namely the PVP group (who underwent conventional PVP) and the PVDP group (who underwent PVP combined percutaneous cement discoplasty). The study started in November 2019 and was finished in March 2021 when the final patient completed at least 12 monthly follow-ups.

**Figure 1:**
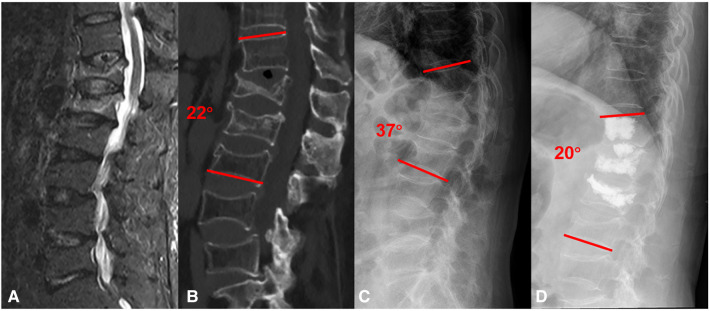
A 76-years old female with vsOVCFs and kyphosis at T12-L1 was treated with PVDP. (**A**) Magnetic resonance imaging (MRI) at admission showed T12-L1 severe compression fracture with kyphosis in preoperative. (**B**) Supine CT scan at admission showed the local kyphosis angle was 22° and T12-L1 with vacuum phenomenon. (**C**) The lateral standing x-ray showed the local kyphosis angle was 37°, the different angulation due to the vertebral and intervertebral collapse (22° and 37°) called the accordion phenomenon. (**D**) Postoperative 12-month x-ray showed that cement was injected in the vertebra and the middle of the T12-L1 intervertebral space and kyphosis was corrected, the local kyphosis angle (20°) was maintained.

Inclusion criteria were: (1) The participants were older than age 60 years; (2) bone mineral density T scores ≤2.5; (3) The diagnosis was thoracolumbar osteoporotic vertebral compression fractures; (4) compression of the vertebral anterior column greater than two-thirds of their original height; (5) Kyphosis with LKA greater than 20°; (6) the accordion phenomenon (14): the different angles of a supine CT scan and a lateral standing x-ray measurement (Figures 1B,C); (7) upper or lower vertebral endplate fracture; (8) the involved the posterior wall of the vertebral body was intact; (9) Informed consent: signed informed consent was obtained from all patients.

Exclusion criteria were: (1) Pathological vertebral fractures caused by spinal tumors, spinal tuberculosis, and spinal infection and so on; (2) Patients with symptoms of nerve roots or spinal cord compression; (3) Patients with a previous history of spinal fusion; (4) A history of abnormal bleeding or coagulation disorder dysfunction; (5) Elderly patients with a history of major illness (such as cardiovascular disease, cancer, or active malignancy) but were tolerant of traditional open surgery.

They were randomly allocated into two groups, namely the PVP group and the PVDP group. The patients were discussed in detail regarding the study and the surgical procedures, and only those who wished to participate were finally included after obtaining informed consent.

### Sample size calculation

Sample size determination A priori power analysis was performed using G-power based on an effect size of 0.8. Considering an alpha level equal to 0.05, and the desired power of 85%. The estimated desired sample was calculated to be 30 patients per group.

### Operative technique

**Figure 2:**
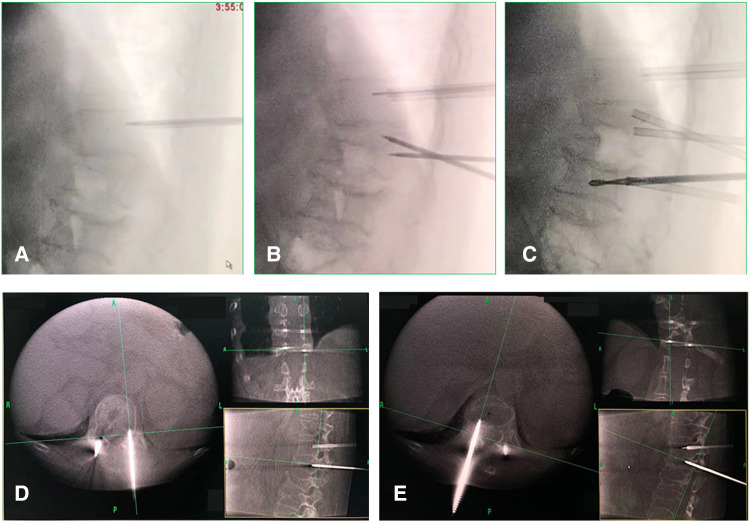
(**A,C**) The puncture needle was inserted into the target area according to the puncture angle and depth measured before the operation. (**B**) The other puncture cannula was inserted into the intervertebral disc space adjacent to the ruptured endplate through the transpedicular access. (**D,E**) Thin-cut CT images were obtained intraoperatively by the O-arm fluoroscopy to confirm puncture needle placement location.

In the PVDP group, PVP and PCD both used a unilateral transpedicular approach into the vertebrae and disc (14). Under general anesthesia, the patient was turned over to a prone position, and the deformity is reduced with a slight closed manipulative. Firstly, under fluoroscopic O-arm guidance, the puncture site was localized into the junction made by the transverse process and the superior articular process and entered the target area according to the puncture angle and depth measured before the operation (Figure 2A). Secondly, the other puncture cannula was inserted into the intervertebral disc space adjacent to the ruptured endplate through the transpedicular access, and the target area was the middle of the intervertebral space (Figure 2B). Thin-cut CT images were obtained intraoperatively by the O-arm fluoroscopy to confirm puncture needle placement location (Figures 2D,E). Third, the bone cement was injected into the vertebrae and the intervertebral disc space, meanwhile, the cannula was also needed to move back to the vertebrae, and the bone cement in the vertebrae could connect the intervertebral disc space and the vertebrae to form a whole. Finally, the PVP group and the adjacent vertebrae performed by prophylactic vertebroplasty used bilateral pedicle puncture, under the guidance of the O-arm, two puncture needles were percutaneously inserted into the vertebrae, reaching about the middle of the vertebrae (Figure 2C). Then, cement was slowly injected into the vertebral body, once cement leakage into the spinal canal or veins was detected, the injection was stopped.

### Parameters observed

Collected and recorded data included patients' age, gender, BMD (T-score), vertebral compression, the amount of bone cement injected, hospital time, and the follow-up duration.

### Study outcomes

The primary outcome was a composite outcome comprised of the visual analog score (VAS) and the Oswestry Disability Index (ODI) associated with the local kyphotic angle (LKA). VAS was used to assess back pain relief, the score is measured between the “no pain” anchor and the patient's mark in a score ruler, providing a range of scores from 0 to 10. A higher score indicates greater pain intensity. ODI was used to assess the quality of life. The ODI assesses ten aspects (pain intensity, personal care, lifting, walking, sitting, standing, sleeping, sex life, social life, and traveling) of daily functions, the elderly can remove their sex life aspect. An ODI of 0%–20%, 21%–40% and 41%–60% indicate minimal, moderate and severe disability, pain remains the main problem in 41%–60% group of patients; patients with an ODI of 61%–80% are severely crippled in function with back pain. Finally, an ODI of 81%–100% indicates that the patients are bed-bound. The LKA was calculated by a measurement called Cobb's method, which measured the angle between the superior endplate of the upper vertebrae and the inferior endplate of the lower vertebrae and was used to assess the degree of local kyphosis of the spine. The three outcomes were recorded 1 day before the operation, 1 day after the operation, and a 1-year follow-up.

As secondary outcomes, we recorded radiological parameters including the disc height anterior (DHA), and disc height posterior (DHP) from lateral plain radiographs preoperatively and at 1 day and final follow-up after surgery. DHA was measured from the anterior points of the disc on lateral plain radiographs. It is used to assess the degree of height loss at the anterior border of the intervertebral space. DHP was measured from the posterior points of the disc on lateral plain radiographs. It is used to assess the degree of height loss at the posterior border of the intervertebral space. A typical case is shown in Figure 1.

### Statistical analysis

SPSS 23.0 statistical software (SPSS, Inc, Chicago, IL, USA) was used for analysis. Quantitative data are expressed as mean ± standard deviation. The paired-sample *t*-test was used to assess a significant difference between preoperative and postoperative continuous variables for both groups. The independent two-sample *t*-test was used to identify a significant difference between the groups. A chi-squared test was used for categorical data. A value of *p* < 0.05 was considered to indicate statistical significance in all analyses.

## Results

### Patient demographic characteristics and baseline data

**Figure 3:**
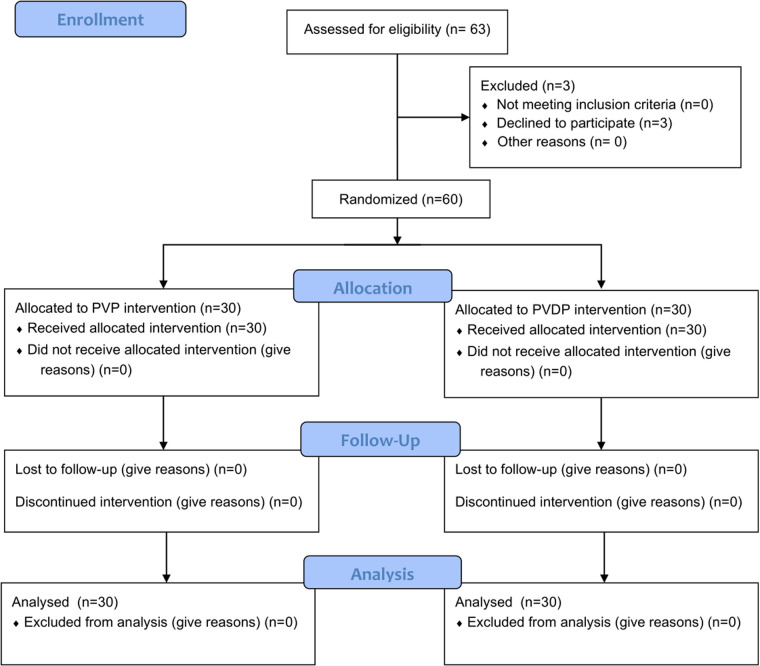
The CONSORT fowchart of the study.

Out of the initial 63 consecutive patients enrolled for the study, 3 patients who would have been eligible for randomization declined to participate. Overall, 60 patients were finally included. Among them, 30 patients each were allocated to the PVP group and PVDP group (Figure 3). Concerning baseline clinical data such as mean age, mean BMD, vertebral compression, hospital days, and follow-up days, there was no significant difference between patients in the PVP group and the PVDP group (Table 1).

**Table 1:** Demographic and clinical characteristics of the patients.

Characteristic	PVP group (*n* = 33)	PVDP group (*n* = 30)	*t*/*χ*^2^	*p* value
Age (years)	76.43 ± 4.95	75.07 ± 7.31	0.848	0.401
Sex (*n*)			0.417	0.519
Male	7	5		
Female	23	25		
BMD (T-score)	−3.19 ± 0.28	−3.17 ± 0.72	−0.094	0.925
Vertebral compression (%)	71.63 ± 5.20	73.47 ± 5.57	−1.318	0.193
Hospital time	3.33 ± 0.48	3.43 ± 0.50	−0.787	0.434
Follow-up duration (months)	27.13 ± 7.98	29.73 ± 7.57	−1.295	0.201

Data are presented as mean ± standard deviation.

### Clinical outcomes

The follow-up results are shown in Table 2. At postoperative follow-up, the VAS and ODI scores of the two groups of patients were significantly lower than those before the operation (*p* < 0.05). However, in the PVDP group, the VAS and ODI scores further decreased to 2.20 ± 0.71 and (22.67 ± 3.52)% at the final follow-up, which was significantly lower than in the PVP group (*p* < 0.05). In both groups, from 1 day to 24 months, no patient was lost to follow-up.

**Table 2:** Primary outcomes results.

Measure	PVP group	PVDP group	*t*	*p* value
VAS score				
Pre-op	7.50 ± 0.86	7.33 ± 0.76	0.796	0.429
1day post-op	3.23 ± 0.82	3.00 ± 0.64	1.229	0.224
Final follow up	3.13 ± 0.82	2.20 ± 0.71	4.703	0.000
Post-op vs. pre-op (mean change, %)	−4.27 (−56.93%)	−4.33 (−59.07%)		
*p*	0.000	0.000		
Final fu vs. post-op (mean change, %)	−0.10 (−3.10%)	−0.8 (−26.67%)		
*p*	0.557	0.000		
ODI score				
Pre-op	70.07 ± 7.47	71.33 ± 5.30	−0.753	0.454
1day post-op	30.89 ± 2.99	29.93 ± 3.18	1.207	0.144
Final follow up	29.04 ± 4.36	22.67 ± 3.52	6.222	0.000
Post-op vs. pre-op (mean change, %)	−39.18 (−55.92%)	−41.40 (−58.04%)		
*p*	0.000	0.000		
Final fu vs. post-op (mean change, %)	−1.85 (−5.99%)	−7.26 (−24.26%)		
*p*	0.006	0.000		
LKA (°)				
Pre-op	36.94 ± 7.12	34.65 ± 9.82	1.036	0.305
1day post-op	29.90 ± 5.12	21.59 ± 3.35	7.427	0.000
Final follow up	32.06 ± 4.60	22.69 ± 5.78	6.944	0.000
Post-op vs. pre-op (mean change, %)	−7.04 (−19.06%)	−13.06 (−37.69%)		
*p*	0.000	0.000		
Final fu vs. post-op (mean change, %)	2.16 (7.22%)	1.10 (5.09%)		
*p*	0.012	0.435		

Data are mean ± standard deviation or number. VAS, visual analogue scale; ODI, Oswestry Disability Index; LKA, the local kyphosis angle. Pre-op vs. post-op and post-op vs. final fu change percentage represented in parentheses.

### Change of the local kyphotic angle

In the PVDP group sagittal plane, the mean thoracolumbar LKA was improved from 34.65° ± 9.82° preoperatively to 21.59° ± 3.35° postoperatively, which was seen to be maintained at the last follow-up (22.69° ± 5.78°). In the PVP group, the average LKA was 36.94° ± 7.12° on admission, decreased to 29.90° ± 5.12° 1 day after the operation, and increased to 32.06° ± 4.60° at the last follow-up. At follow-up of 1 day and 24 months, the kyphotic Cobb angles of the PVDP group were significantly lower than that of the PVP group (*p* < 0.05) and the mean change was significantly lower in the PVDP group at the last follow-up (LKA mean change: 2.16° vs. 1.10°, 7.22% vs. 5.09%, Table 2).

### Radiographic outcomes

Compared with those on pre-operation, in the PVDP group, the DHA changed from 5.62 ± 1.46 to 7.01 ± 1.04 mm, the DHP changed from 4.16 ± 0.79 to 4.63 ± 0.66 mm, which were seen to be maintained at the final follow-up (DHA: 6.61 ± 1.43 mm; DHP: 4.37 ± 1.08 mm; *p* > 0.05). However, in the PVP group, the DHA decreased from 6.22 ± 1.65 mm 1 day after operation to 5.36 ± 1.16 mm at the last follow-up (*p* < 0.05) and the DHP decreased from 4.05 ± 0.41 to 3.83 ± 0.63 mm. The DHA and DHP for the patients in the PVP group were each significantly lower than in the PVDP group (*p* < 0.05). In both parameters, the change was significantly lower in the PVDP group at the last follow-up (Table 3).

**Table 3:** Results of the radiological measurements.

Measure	PVP group	PVDP group	*t*	*p* value
DHA (mm)				
Pre-op	6.18 ± 1.55	5.62 ± 1.46	1.438	0.157
1day post-op	6.22 ± 1.65	7.01 ± 1.04	−2.112	0.039
Final follow up	5.36 ± 1.16	6.61 ± 1.43	−3.707	0.000
Post-op vs. pre-op (mean change, %)	0.04 (0.65%)	1.39 (24.73%)		
*p*	0.910	0.000		
Final fu vs. post-op (mean change, %)	−0.86 (−13.83%)	−0.40 (−5.71%)		
*p*	0.002	0.201		
DHP (mm)				
Pre-op	4.30 ± 0.90	4.16 ± 0.79	0.586	0.560
1day post-op	4.05 ± 0.41	4.63 ± 0.66	−4.116	0.000
Final follow up	3.83 ± 0.63	4.37 ± 1.08	−2.356	0.022
Post-op vs. pre-op (mean change, %)	−0.25 (−5.81%)	0.47 (11.30%)		
*p*	0.337	0.003		
Final fu vs. post-op (mean change, %)	−0.22 (−5.43%)	−0.26 (−5.62%)		
*p*	0.094	0.299		
Cement volume (ml)	4.08 ± 0.68	5.12 ± 0.64	−6.052	0.000

Data are mean ± standard deviation or number. DHA, disc height anterior; DHP, disc height posterior. Pre-op vs. post-op and post-op vs. final fu change percentage represented in parentheses.

### Complications

The amount of cement injected was 4.08 ± 0.68 ml in the control group, and more cement was injected in the PVDP group (5.12 ± 0.64, *p* < 0.05) (Table 3). During the follow-up period, the rate of cement leakage in our study was 23.3%. Asymptomatic cement leakages occurred in 3 cases and leaked into the paravertebral space (*n* = 3) in the PVDP group. In the control group, cement leakages were found in 9 cases with 11 locations distributed among the intervertebral disc space (*n* = 7), and paravertebral space (*n* = 4).

## Discussion

The treatment of vsOVCFs remains controversial. Conservative treatment is invalid for the pain relief of vsOVCF. For elderly patients, it often leads to various complications such as kyphosis, pulmonary infection, deep vein thrombosis, and bedsores. These patients are also not able to tolerate osteotomy and internal fixation due to the large surgical trauma, difficulty in fixation, and high operative complications. PVP has been widely considered the most suitable approach for older patients unsuitable candidates for open surgery. However, vsOVCFs have always been recognized in the past as an absolute or relative contraindication due to the high risk of cement leakage and surgical technical difficulties.

With the development of imaging and minimally invasive surgical technology, PVP has gradually been applied to the treatment of vsOVCFs. The previous studies (15) have demonstrated that patients with vsOVCFs had immediate pain relief after PVP surgery. However, the injured vertebrae in patients with vsOVCFs have been a significant collapse and were usually accompanied by the upper or lower vertebral endplate fracture, therefore, patients were at a high risk of cement leakage. Nieuwenhuijse et al. (16) reported that cement leakage occurred in 91.9% of patients with vsOVCFs receiving PVP treatment. Very severe vertebral compression fractures lead to severe reduction of intervertebral space height and kyphosis deformity. Meanwhile, Zhao et al. (17) have shown that the acute trauma on intervertebral discs and the cement leakage into the disc are both considered key factors in acceleration of disc degeneration, which will result in a decrease in the intervertebral space height and an increase in the local kyphotic angle. In vsOVCFs, PVP treatment can relieve pain and improve the quality of life, but the maintenance of intervertebral space height and the correction of kyphosis are limited. The reason for this result may be that the disc injury may result in the acceleration of disc degeneration, and the intervertebral space leakage of bone cement will further accelerate the degeneration and decrease the height of the intervertebral disc, leading to the loss of kyphotic correction.

In this study, back pain symptoms of vsOVCFs patients after PVP often cannot be completely relieved. Endplate disc injury and kyphosis are some of the causes of pain, and segmental intervertebral instability is also an important reason. Some scholars (10) believe that the local kyphosis of the spine fracture exceeds 20°, and the compression degree of the anterior column of the diseased vertebral is more than 50%, it can be considered an unstable fracture. Meanwhile, due to the injury of the intervertebral disc and the lack of formal conservative treatment, severe degeneration of the intervertebral disc or even the appearance of a vacuum phenomenon should also be regarded as a sign of instability (14), and the different angles of a supine CT scan and a lateral standing x-ray measurement are called the accordion phenomenon (Figures 1B,C). Percutaneous cement discoplasty (PCD) was an emerging technology proposed by Varga (13) first in 2014, and the principle is to use the cement injected into the disc as a simple interbody fusion to the therapeutic effect of achieving back pain relief caused by dynamic foraminal stenosis and vacuum phenomenon in the intervertebral disc. Carlos Sola studied that PCD can be considered as an alternative minimal invasive therapeutic modality for the treatment of advanced degenerative disc disease like a spinal deformity or degenerative spondylolisthesis, with satisfactory clinical effects. Li et al. (18) made an effective and accurate finite element PCD model to simulate the motion of the spine. Their study demonstrated that an increase in friction or even a fusion between cement and endplate contributed to higher stability after the injection of bone cement into the disc. Meanwhile, due to the supportive effects of bone cement, the bone cement is relatively stable after PCD and does not squeeze the annulus fibrosus or dislocate from a weak point. Recently, Xue et al. (19) have proposed that percutaneous kyphoplasty (PKP) combined with PCD to treat severe thoracolumbar vsOVCFs has also achieved good results. They shifted the working channel to insert the puncture needle into the upper intervertebral disc through the adjacent endplate and then injected the cement. However, for very severe osteoporotic vertebral compression fractures with less compression in the supine position than in the standing position, there is a spatial basis for expansion and reduction (20, 21). Balloon dilation follows the path of least resistance, but surgeons cannot determine the location and direction of balloon expansion during PKP procedures. When the collapse rate of the vertebral body is very severe, the backward dilation of the balloon will squeeze the posterior trabecular bone or the posterior wall of the vertebral body, thereby aggravating the compression of the spinal cord (22). Meanwhile, the uncontrollable position of Balloon dilation will lead to the poor reduction of endplate fractures (23). Takahashi et al. (24) also conducted a risk factor analysis of revision after PKP and found that endplate destruction increased the revision rate by 5-fold. Therefore, we consider that the surgical methods of PKP combined with PCD are not very suitable for very severe vsOVCFs disease. We believe that unnecessary vertebral body damage will occur during this transfer process, and the expansion of the balloon may cause secondary damage to the endplate and the posterior wall of the vertebral body (23, 25).

In our study, we compare the clinical and radiological outcomes of patients with vsOVCFs in the PVP group and the PVDP group to prove whether the PVP combined with PCD surgery is more suitable for the treatment of vsOVCFs. To the best of our knowledge, this is the first study of its kind. The average VAS score and ODI score in both groups decreased after surgery and at the final follow-up, but more so for patients in the PVDP group than those in the PVP group. These findings suggested that both PVDP and PVP could sustain pain relief, but PVDP outperformed PVP in patients with vsOVCFs. The better improvement of these symptoms might be attributed to the stabilization of the segmental intervertebral after PVDP. At the follow-up of 1 day after surgery, the LKA was significantly decreased and DHA was significantly increased for patients in the PVDP group, which was seen to be maintained at the last follow-up, however, the LKA was increased and DHA was decreased in the PVP group at the last follow-up. The significant improvements might be attributable to the effectively supporting effect of the anterior column maintained by the bone cement in disc space after PVDP.

Previous related studies have not reported whether PCD will increase the risk of adjacent vertebral fractures (AVF). However, most scholars (26, 27) currently believe that preoperative severe kyphosis deformity, stiffness enhancement of the treated vertebra, and cement leakage in the intervertebral space increase the risk of AVF. Since all patients in the present study were diagnosed with primary osteoporosis, prophylactic vertebroplasty was performed on adjacent vertebrae undergoing PCD to reduce the risk of AVF.

The limitations of this study deserve mentioned. Firstly, in the previous study, the disc vacuum phenomenon is an indication for PCD. This study defines the indication as severe degeneration of the intervertebral disc and the disc vacuum phenomenon because we believe that the dispersion of bone cement in the intervertebral disc may be also related to the density of the intervertebral disc. It may be necessary to further compare the two groups. Secondly, in this study, severe local kyphosis can be considered as spinal instability. It would be more meaningful if the flexion-extension radiograph of the spine can be performed under the premise of analgesia. Beyond that, it is a preliminary retrospective study with a relatively small number of patients eligible for inclusion criteria and a relatively short follow-up period. Future investigations with larger samples are required to verify the present results.

## Conclusion

Due to the increased risk of perioperative complications, elderly patients with multiple comorbidities suffering from vsOVCFs are generally not suitable for long-term open surgery, we established that PVDP may be a feasible and effective technique for the treatment of very severe OVCFs, which could effectively relieve pain, improve life quality, restore intervertebral height and improve kyphosis.

## Data Availability

The raw data supporting the conclusions of this article will be made available by the authors, without undue reservation.
