# Supplementary figures and images for: Percutaneous vertebral-disc plasty for thoracolumbar very severe osteoporotic vertebral compression fractures: A randomized controlled study
Source: Front Surg. 2022 Oct 19;9:1010042. doi: 10.3389/fsurg.2022.1010042 (PMC9627294; doi:10.3389/fsurg.2022.1010042)

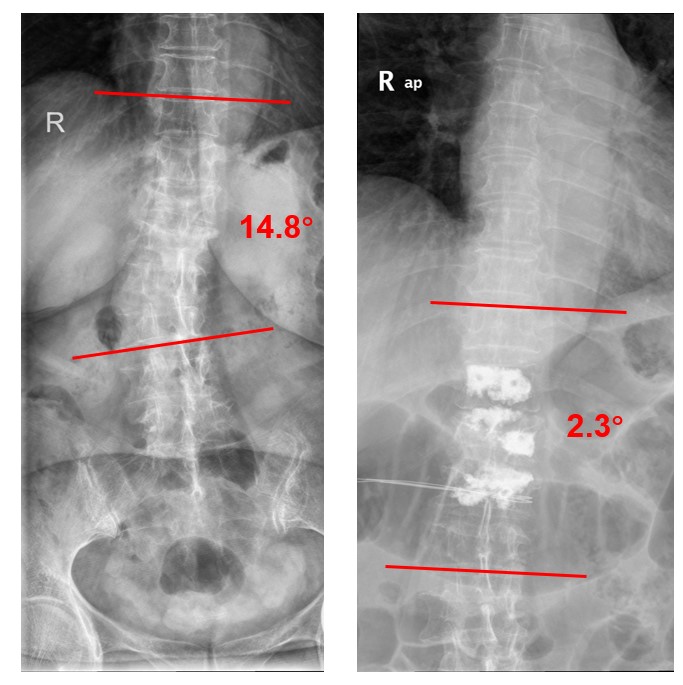

Supplement: Supplementary file 1 [file Image1.jpeg]
